# Case report: Clinical utility and limitations of high-frequency ultrasound for monitoring sonidegib response in nodular basal cell carcinoma: a three-case series

**DOI:** 10.3389/fonc.2026.1945673

**Published:** 2026-10-07

**Authors:** Dandan He, Hailiang Tao, Ke Bian, Junzhu Xu, Tao Wang

**Affiliations:** 1 Department of Ultrasound, Hangzhou Third People’s Hospital, Hangzhou Third Hospital Affiliated to Zhejiang Chinese Medical University, Hangzhou, China; 2 Department of Dermatology, Hangzhou Third People’s Hospital, Hangzhou Third Hospital Affiliated to Zhejiang Chinese Medical University, Hangzhou, China

**Keywords:** basal cell carcinoma, high-frequency ultrasound, imaging complete response, nodular type, pathological response, sonidegib

## Abstract

**Background:**

Sonidegib, a Hedgehog pathway inhibitor, can induce clinical and imaging remission in locally advanced basal cell carcinoma (BCC). Residual deep tumor may persist after malignant dermoscopic and ultrasound features disappear. This case series assessed the utility and limitations of 20–30 MHz high-frequency ultrasound (HFUS) for confirming complete imaging remission after sonidegib.

**Cases Presentation:**

Three patients with nodular BCC received sonidegib 200 mg daily (October 2024–May 2026). Two achieved complete imaging remission, defined as resolution of malignant imaging features with residual non-malignant hypoechoic areas allowed, and underwent surgical resection with histopathological correlation; the third declined surgery. Baseline ultrasound was unavailable, so findings were compared with the earliest 3-4-month follow-up.

**Results:**

Response involved lesion shrinkage and loss of intratumoral blood flow. Serial imaging showed gradual resolution of hypoechoic nodules with vascularity on HFUS and blue-gray nests with arborizing vessels on dermoscopy, leaving avascular scar-like structures. Preoperative HFUS residual hypoechoic areas were correlated with histopathology, which showed chronic inflammatory infiltration (case 1) and mild dermal fibrosis (case 2), with no residual tumor. The third patient’s malignant features regressed after six months.

**Conclusions:**

In this small case series, HFUS monitored deeper tissue changes after targeted therapy. In two histopathologically confirmed patients, complete imaging remission on HFUS plus dermoscopy corresponded to pathological complete response; however, given the small sample and histopathological confirmation in only two patients, this is a possible correspondence rather than evidence of true predictive capability. Post-treatment hypoechoic areas may reflect inflammation, fibrosis, or residual tumor, limiting specificity. Imaging-based complete response should be confirmed by biopsy or excision when feasible. Surgery remains the gold standard; non-surgical patients require close follow-up.

## 1 Introduction

Basal cell carcinoma (BCC) is the most common malignant skin tumor. For locally advanced or surgery-ineligible BCC, the oral Hedgehog pathway inhibitor sonidegib is an important treatment option (1). Response is generally assessed by clinical examination and dermoscopy, with disappearance of characteristic features such as blue-gray ovoid nests and arborizing vessels regarded as response (2, 3). However, dermoscopy mainly visualizes the epidermis and superficial dermis and cannot reliably detect residual tumor, fibrosis, or inflammation in deeper tissues (4). High-frequency ultrasound (HFUS; 20–30 MHz) visualizes full skin thickness and superficial subcutaneous tissue and can assess lesion dimensions, margins, echogenicity, and vascularity (5, 6). We report three patients with nodular BCC treated with sonidegib who underwent serial standardized dermoscopy and HFUS. Two achieved complete imaging remission and underwent surgery, enabling correlation of preoperative HFUS findings with serial histopathological sections; the third achieved partial imaging remission but declined surgery. Response definitions and HFUS criteria are briefly described at the beginning of the Case Presentation.

In this report, complete imaging remission was defined as disappearance of malignant sonographic and dermoscopic features characteristic of BCC, rather than complete absence of any residual hypoechoic area. Ultrasound criteria included: (i) resolution of irregular hypoechoic tumor mass with punctate hyperechoic foci and posterior acoustic enhancement; (ii) absence of intralesional and perilesional Doppler signals; and (iii) residual hypoechoic areas that were avascular, lacked hyperechoic foci, and did not enlarge on serial HFUS, consistent with post-treatment fibrosis, scar, or chronic inflammation. Progressive enlargement of a hypoechoic area on follow-up was considered suspicious for residual or recurrent disease. Histopathological confirmation remained the reference standard.

## 2 Case description

### 2.1 Case 1

A 73-year-old man presented with a 6-month history of an ulcerated black papule on the right lower eyelid and medial canthus. Examination showed a 10 × 10 mm well-demarcated lesion extending into subcutaneous tissue without orbicularis oculi or orbital invasion (**Figure 1A**). Dermoscopy showed large blue-gray ovoid nests, blue-gray dots/globules, ulceration, and arborizing telangiectasias (**Figure 1B**). Incisional biopsy confirmed nodular BCC. Given the tumor location near the globe and lacrimal system, surgical resection was considered challenging and risky, with possible ectropion or lacrimal duct injury. A multidisciplinary team (MDT) comprising Dermatology, Plastic Surgery, Ophthalmology, and Oncology recommended sonidegib 200 mg once daily. No baseline ultrasound was performed before sonidegib initiation; serial changes were therefore referenced to the 4-month scan (**Figure 1C**). HFUS showed progressive regression of a dermal hypoechoic nodule, with a 2.0 × 1.0 mm avascular residual area without hyperechoic foci by 9 months (**Figure 1D**). Grade 1–2 dysgeusia, decreased appetite, and muscle cramps occurred after 6 months and were managed symptomatically. Surgical excision after 9 months showed chronic dermal inflammatory infiltrates without residual tumor cells, confirming pCR (**Figure 1E**). The residual hypoechoic area corresponded to inflammation rather than BCC. No recurrence was observed at short-term follow-up. A detailed timeline of the episode of care is shown in (**Table 1**).

**Figure 1:**
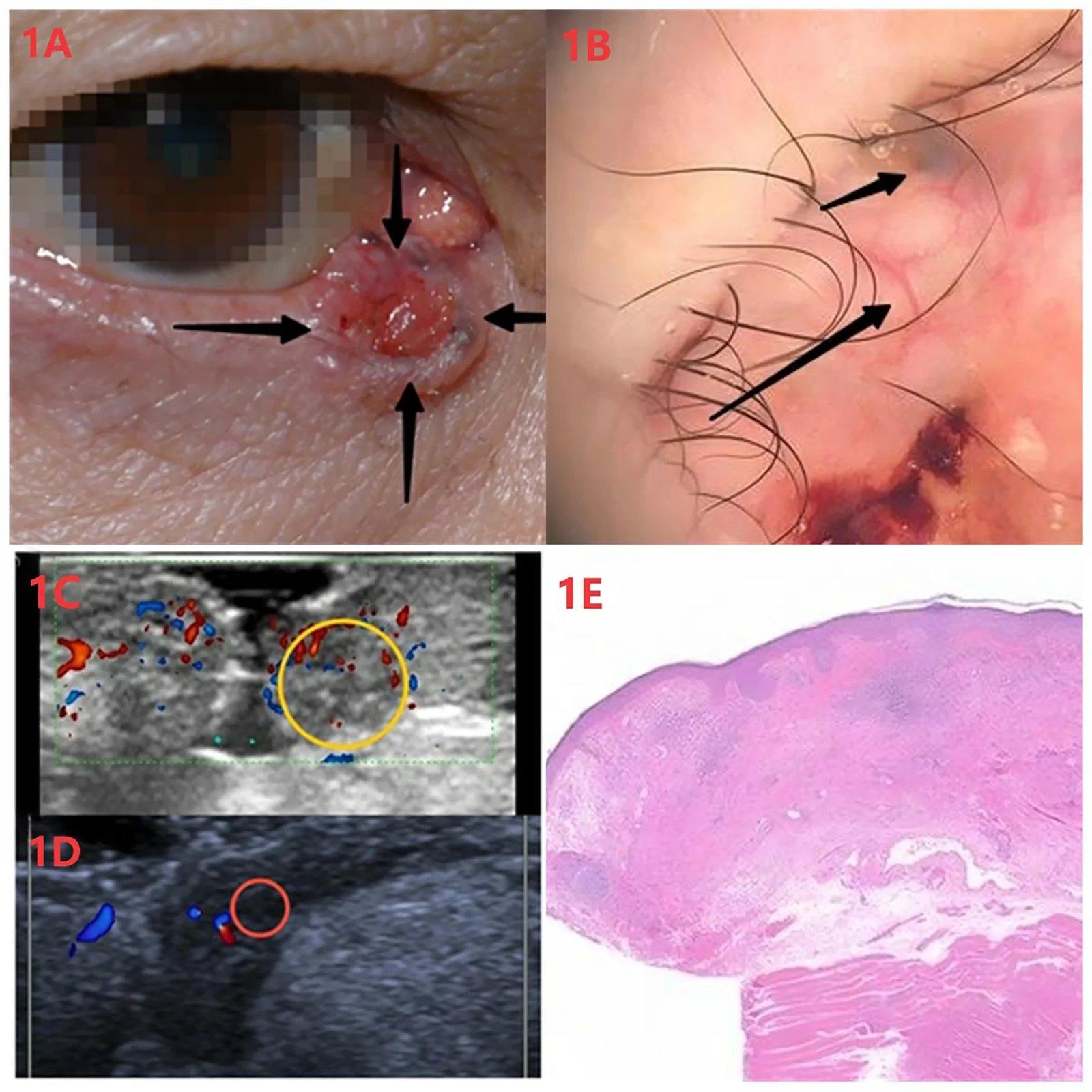
Clinical、dermatoscope and imaging and pathological features of the 73-year-old man with a black papule on the right lower eyelid. **(A)** Clinical image showing the clear tumor extent (the black arrows). **(B)** Dermoscopy image (Polarized-light dermoscopy at 30× magnification showing an infiltrative pattern) before treatment:the long black arrow indicates the dendritic vasculature of BCC, the short black arrow indicates the large blue-gray ovoid nests. **(C)** HFUS image of Solideji after 4 months of treatment:yellow solid linea mark area indicates the tumor and blood flow signals. **(D)** HFUS image of Solideji after 9 months of treatment:red solid linea mark area indicates residual hypoechoic area completely devoid of flow signals or hyperechoic foci, suggesting tumor involution. **(E)** Hematoxylin and eosin staining reveals a normal epidermis with patchy superficial and mid-dermal infiltrates of lymphocytes, histiocytes, and a few plasma cells, no residual tumor cells were identified.

**Table 1:** Timeline of clinical, dermoscopic, ultrasound, therapeutic, and histopathological findings in Case 1.

Time point	Clinical / therapeutic events	Dermoscopy	HFUS	Histopathology / outcome
6 months before presentation	Black papule on right lower eyelid and medial canthus	——	——	——
4 months before presentation	Ulceration developed	——	——	——
Initial presentation	73-year-old man; 10 × 10 mm well-demarcated lesion extending into subcutaneous tissue; no invasion of orbicularis oculi or orbit; MDT recommended sonidegib 200 mg once daily	Large blue-gray ovoid nests, multiple blue-gray dots and globules, surface ulceration, arborizing telangiectasias	No baseline ultrasound performed	Incisional biopsy (4 mm diameter, 2 mm depth) confirmed nodular BCC
Sonidegib initiation	Oral sonidegib 200 mg once daily started	——	——	——
4 months	Clinically reduced lesion size	Monitored	Earliest HFUS: well-defined dermal hypoechoic nodule 4.4 × 3.7 × 3.3 mm; punctate hyperechoic foci; linear intralesional blood flow	——
6 months	Grade 1-2 dysgeusia, decreased appetite, muscle cramps; managed symptomatically	Monitored	Residual hypoechoic area 2.0 × 1.4 mm; ill-defined margins; loss of round homogeneous echo pattern; punctate blood flow; few punctate hyperechoic foci	——
9 months	Surgical excision	Monitored	Residual hypoechoic area 2.0 × 1.0 mm; no flow signals; no hyperechoic foci; suggestive of tumor involution	Postoperative histopathology: normal epidermis; patchy superficial and mid-dermal infiltrates of lymphocytes, histiocytes, and a few plasma cells; no residual tumor cells; pCR. Residual hypoechoic area corresponded to chronic inflammatory infiltrates
Short-term follow-up	No recurrence	——	——	——

HFUS, high-frequency ultrasound; BCC, basal cell carcinoma; MDT, multidisciplinary team; pCR, pathologic complete response. No baseline ultrasound was available before sonidegib initiation; the 4-month HFUS was the earliest available examination.

### 2.2 Case 2

A 47-year-old woman presented with a 2-year history of a black papule on the right nasal ala, which had enlarged over the past year with irregular hyperpigmentation. Examination revealed a 15 × 15 mm lesion extending into subcutaneous tissue but sparing underlying muscle and nasal bone (**Figure 2A**). Dermoscopy showed large blue-gray ovoid nests, blue-gray dots/globules, maple leaf-like areas, spoke-wheel structures, ulceration, and arborizing telangiectasias (**Figure 2B**). Incisional biopsy confirmed BCC. Given its location on the right nasal ala (1.5 cm), surgery was challenging. The thin skin and delicate alar cartilage meant wide excision could cause alar collapse, nasal deformity, and nasal valve impairment, affecting cosmesis and respiratory function. A MDT (Dermatology, Plastic Surgery, Otolaryngology, Oncology) recommended sonidegib 200 mg once daily. No baseline ultrasound was performed before sonidegib initiation; serial changes were referenced to the 3-month scan. HFUS showed a large ill-defined hypoechoic dermal/subcutaneous nodule at 3 months (**Figure 2C**), partial regression at 6 months, and complete disappearance of intralesional flow and hyperechoic foci by 8 months (**Figure 2D**). Leg muscle soreness occurred at 1 month, and ageusia and diffuse alopecia at 3 months. Excision after 8 months showed fibrotic remodeling without residual tumor cells, confirming pCR (**Figure 2E**). No recurrence was observed at short-term follow-up.

**Figure 2:**
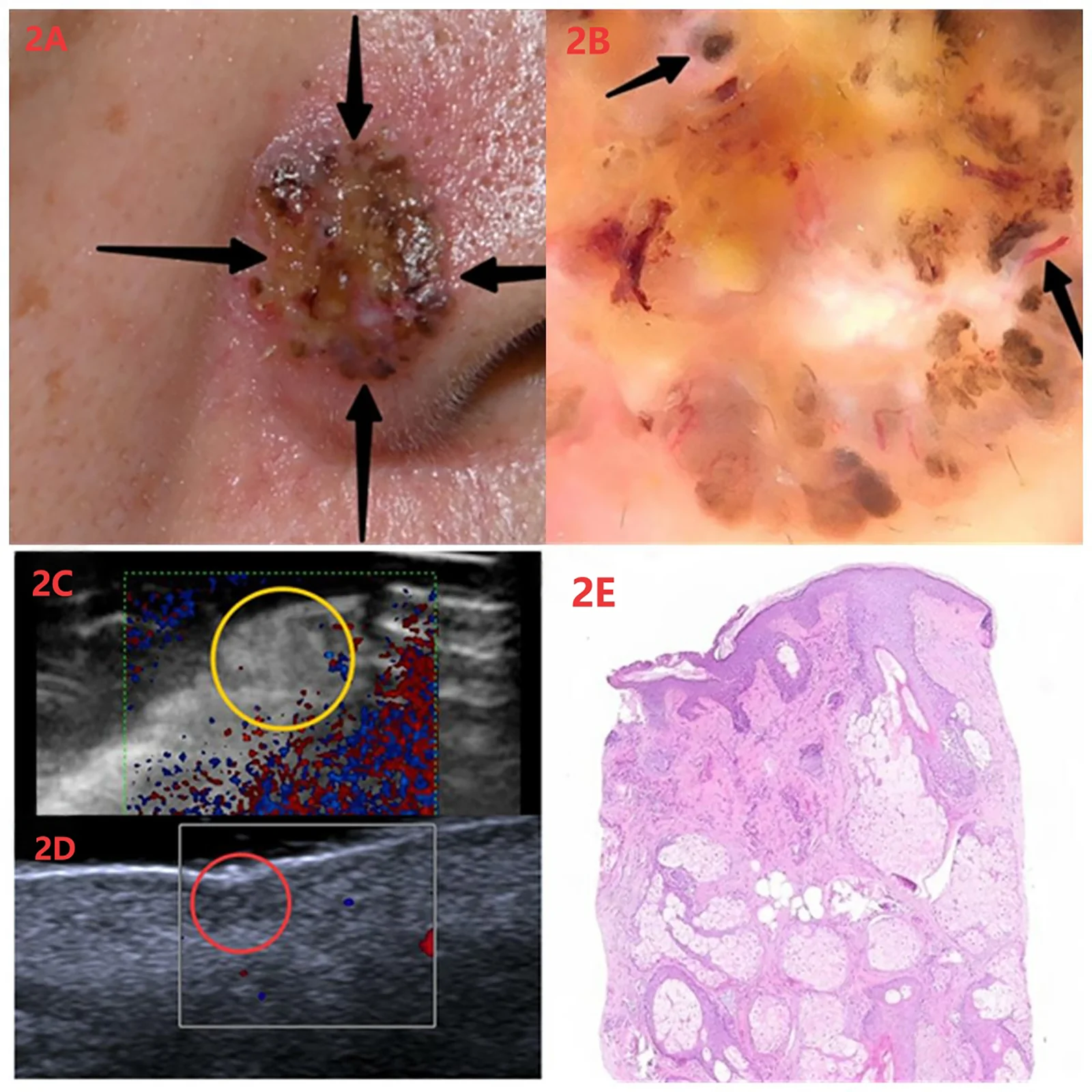
Clinical、dermatoscope and imaging and pathological features of the 47-year-old woman with a black papule on the right nasal ala. **(A)** Clinical image showing the clear tumor extent (the black arrows). **(B)** Dermoscopy image (Polarized-light dermoscopy at 20× magnification showing an infiltrative pattern) before treatment:the long black arrow indicates the dendritic vasculature of BCC, the short black arrow indicates the large blue-gray ovoid nests. **(C)** HFUS image of Solideji after 3 months of treatment:yellow solid linea mark area indicates the tumor and blood flow signals. **(D)** HFUS image of Solideji after 8 months of treatment:red solid linea mark area indicates residual hypoechoic area completely devoid of flow signals or hyperechoic foci, suggesting tumor involution. **(E)** Hematoxylin and eosin staining reveals a normal epidermis with mild dermal fibroblast proliferation and sparse perivascular lymphocytic infiltrates, no residual tumor cells were identified.

A detailed timeline of the episode of care is shown in (**Table 2**).

**Table 2:** Timeline of clinical, dermoscopic, ultrasound, therapeutic, adverse event, and histopathological findings in Case 2.

Time point	Clinical / therapeutic events	Dermoscopy	HFUS	Adverse events	Histopathology / outcome
2 years before presentation	Black papule on right nasal ala	——	——	——	——
Over the past year	Enlargement with irregular hyperpigmentation	——	——	——	——
Initial presentation	47-year-old woman; 15 × 15 mm lesion extending into subcutaneous tissue but sparing underlying muscle and nasal bone; MDT recommended oral sonidegib 200 mg once daily	Large blue-gray ovoid nests, blue-gray dots and globules, maple leaf-like areas, spoke-wheel structures, ulceration, arborizing telangiectasias	No baseline ultrasound performed	——	Incisional biopsy confirmed BCC
Sonidegib initiation	Oral sonidegib 200 mg once daily started	——	——	——	——
1 month	Continued sonidegib	——	——	Leg muscle soreness	——
3 months	Continued sonidegib	Monitored	Earliest HFUS: large, ill-defined hypoechoic dermal/subcutaneous nodule 10.3 × 3.9 × 8.2 mm; punctate hyperechoic foci; intralesional blood flow, indicating viable deep tumor	Ageusia and diffuse alopecia	——
6 months	Continued sonidegib	Monitored	Nodule decreased to 5.3 × 2.0 × 7.2 mm; reduced punctate flow and hyperechoic foci; consistent with partial regression	——	——
8 months	Surgical excision	Monitored	Lesion 4.9 × 1.8 × 6.6 mm; blurred margins; all intralesional blood flow and hyperechoic foci disappeared, suggesting complete involution and fibrotic conversion	——	Normal epidermis; mild dermal fibroblast proliferation; sparse perivascular lymphocytic infiltrates; no residual tumor cells; pCR. Residual hypoechoic area corresponded to fibrotic remodeling, not viable carcinoma
Short-term follow-up	No recurrence	——	——	——	Recovered well

HFUS, high-frequency ultrasound; BCC, basal cell carcinoma; MDT, multidisciplinary team; pCR, pathologic complete response. No baseline ultrasound was available before sonidegib initiation; the 3-month HFUS was the earliest available examination. The residual hypoechoic area at 8 months was avascular, lacked hyperechoic foci, and corresponded histopathologically to fibrotic remodeling rather than viable tumor.

### 2.3 Case 3

A 78-year-old woman presented with an 8-year history of black papules on the nasal tip that had enlarged over 12 months with irregular hyperpigmentation. Her history included untreated lung cancer and pituitary adenoma. Examination showed a 15 × 15 mm lesion extending into subcutaneous tissue but sparing the underlying muscle and nasal bone (**Figure 3A**). Dermoscopy showed large blue-gray ovoid nests, blue-gray dots/globules, maple leaf-like and spoke-wheel areas, ulceration, and arborizing telangiectasias (**Figure 3B**). Incisional biopsy confirmed nodular BCC. Because of the nasal tip location, risk of a full-thickness defect and disfigurement, advanced age, comorbidities, and the patient’s refusal of surgery, A MDT(Dermatology, Plastic Surgery, Otolaryngology, Oncology)recommended sonidegib 200 mg once daily, consistent with its approved indication for locally advanced BCC in patients who are not surgical candidates.

**Figure 3:**
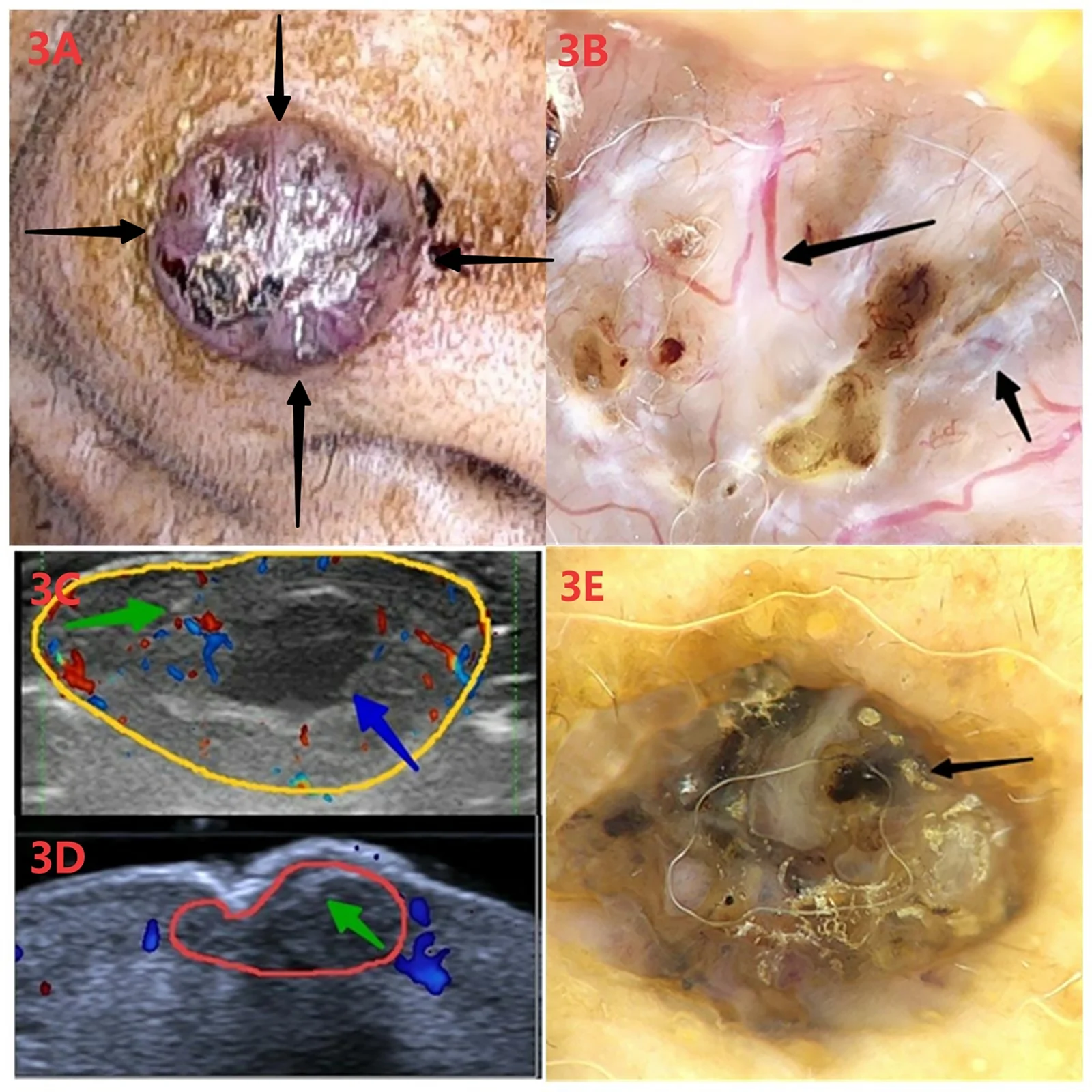
Clinical、dermatoscope and imaging features of the 78-year-old woman with a black papules on the nasal tip. **(A)** Clinical image showing the clear tumor extent (the black arrows). **(B)** Dermoscopy image (Polarized-light dermoscopy at 40× magnification showing an infiltrative pattern) before treatment:the long black arrow indicates the dendritic vasculature of BCC, the short black arrow indicates the large blue-gray ovoid nests. **(C)** HFUS image before treatment:yellow solid linea mark area indicates the tumor and blood flow signal, The blue arrow indicates a anechoic area, while the green arrow denotes the punctate hyperechoic foci. **(D)** HFUS ultrasound image of Solideji after 6 months of treatment:red solid linea mark area indicates residual hypoechoic area, the hypoechoic areas and blood flow signals have disappeared, while punctate hyperechoic foci remained (the green arrow). **(E)** Dermoscopy image (Polarized-light dermoscopy at 30× magnification showing an infiltrative pattern) of Solideji after 6 months of treatment:the short black arrow indicates the large blue-gray ovoid nests, the dendritic vasculature of BCC have disappeared.

Baseline HFUS (**Figure 3C**) showed a well-defined hypoechoic nodule (11.9 × 7.1 × 11.3 mm) with anechoic areas, punctate hyperechoic foci, and abundant linear intralesional flow. At 6 months (**Figure 3D**), the nodule had decreased to 7.7 × 3.6 × 8.3 mm with blurred margins; anechoic areas and all intralesional flow had disappeared, while punctate hyperechoic foci persisted, suggesting fibrotic or calcific remnants without viable tumor perfusion. Grade 1–2 decreased appetite and dysgeusia occurred at 1 month, and diffuse alopecia at 3 months; these were managed symptomatically without dose reduction. The patient declined surgery and continued sonidegib under surveillance. At 6 months, dermoscopy showed residual pigmented structures but no arborizing vessels (**Figure 3E**), and HFUS persistently showed no deep intralesional blood flow, suggesting sustained suppression of tumor viability; however, histopathological confirmation was unavailable because surgery was declined. Follow-up is ongoing.

## 3 Diagnostic assessment & therapeutic intervention

### 3.1 Diagnostic assessment

All three patients underwent initial diagnostic evaluation including physical examination and dermoscopy. Incisional biopsy (4 mm diameter, 2 mm depth) at the lesion edge confirmed nodular basal cell carcinoma (BCC) in each.

HFUS was used for longitudinal monitoring in all three, though timing varied:

- Case 1: 4, 6, and 9 months after treatment initiation.
- Case 2: 3, 6, and 8 months after treatment initiation.
- Case 3: baseline (before biopsy and treatment) and 6 months of therapy.

HFUS was performed using two systems: a Siemens Medical Solutions USA system with an 18H6 linear array probe (6–21 MHz) and an Esaote MyLab ClassC system (Italy) with an SL3116 linear array probe (12–25 MHz). Intralesional vascular signals were evaluated with color Doppler. Because this case series was retrospective, PRF/scale, gain, and wall filter were not uniformly recorded; only available settings are reported, which is a limitation. HFUS parameters included lesion dimensions (maximum diameter, depth, volume), echogenicity, margin characteristics (well- vs. ill-defined), internal architecture (anechoic areas, punctate hyperechoic foci), and intralesional vascularity (presence and abundance of flow signals).

Dermoscopy was performed at baseline and each follow-up using standardized polarized dermoscopy. Key features assessed included large blue-gray ovoid nests, blue-gray dots and globules, maple leaf-like areas, spoke-wheel structures, ulceration, and arborizing telangiectatic vessels.

MDT discussions were conducted for each case, with specialists from Dermatology, Plastic Surgery, Ophthalmology/Otolaryngology, and Oncology jointly reviewing clinical, imaging, and pathological findings to guide therapeutic decision-making.

### 3.2 Rationale for sonidegib therapy

In all three cases, surgical resection was deemed suboptimal or contraindicated due to anatomical vulnerability, functional risks, and/or patient-specific factors.

- In Case 1, the lesion was located at the right lower eyelid and medial canthus, in close proximity to the globe and lacrimal system. Accordingly, wide local excision posed substantial risks of ectropion and lacrimal duct injury.
- In Case 2, the lesion involved the right nasal ala (1.5 cm in diameter). Given the thin skin and delicate alar cartilage, conventional excision with adequate margins threatened alar collapse, nasal valve dysfunction, and significant cosmetic deformity affecting both appearance and respiratory function.
- In Case 3, the lesion was situated at the nasal tip. Surgical resection would have created a large structural defect, leading to tip flattening and contour deformity. Moreover, the patient’s advanced age (78 years), significant comorbidities (untreated lung cancer and pituitary adenoma), and explicit refusal of surgery collectively precluded operative intervention.

Following MDT deliberations, oral sonidegib 200 mg once daily was initiated in all three patients until disease progression, unacceptable toxicity, or completion of the planned treatment course—as neoadjuvant therapy in Cases 1 and 2 (with planned subsequent surgery), and as definitive systemic therapy in Case 3 (with ongoing surveillance). Serial HFUS and dermoscopy were employed to monitor therapeutic response at regular intervals. Summary of therapeutic outcomes (**Table 3**).

**Table 3:** Summary of therapeutic outcomes.

Case	Age /sex	Lesion site	Baseline size	Treatment intent	Treatment duration	Best radiologic response(HFUS)	Adverse events	Pathologic outcome	Follow-up status
1	73/M	Right lower eyelid & medial canthus	10 × 10 mm	Neoadjuvant	9 months	Near-complete regression (2.0 × 1.0 mm, no flow)	Dysgeusia, decreased appetite, muscle cramps (Grade 1-2)	pCR (confirmed post-excision)	Recurrence-free at short-term follow-up
2	47/F	Right nasal ala	15 × 15 mm	Neoadjuvant	8 months	Near-complete regression (4.9 × 1.8 × 6.6 mm, no flow)	Leg soreness (month 1), ageusia & alopecia (month 3) (Grade 1-2)	pCR (confirmed post-excision)	Recurrence-free at short-term follow-up
3	78/F	Nasal tip	15 × 15 mm (11.9 × 7.1 × 11.3 mm on HFUS)	Definitive systemic therapy	6 months (ongoing)	Partial regression (7.7 × 3.6 × 8.3 mm, no flow)	Decreased appetite & dysgeusia (month 1), noticeable alopecia (month 3) (Grade 1-2)	Pending (surgery declined)	Ongoing surveillance; no clinical progression

HFUS measurements represent maximum lesion diameters (anteroposterior × width × depth). pCR, pathologic complete response; HFUS, high-frequency ultrasound.

## 4 Patient perspective

Sonidegib can shrink locally advanced basal cell carcinoma before surgery, offering hope of preserving appearance and function. For patients who cannot or choose not to undergo surgery, it provides a systemic option. HFUS and dermoscopy allow repeated, noninvasive monitoring. However, side effects—muscle soreness, taste loss or change, hair loss, and reduced appetite—are mostly mild to moderate. More concerning, imaging remission does not prove cancer absence. A residual hypoechoic area may be inflammation, fibrosis, or viable tumor, which ultrasound cannot reliably distinguish. Without surgery, pathological confirmation is impossible, leaving uncertainty. Surgery remains the gold standard, and close long-term follow-up is essential. Sonidegib is valuable for tumor control and organ preservation but cannot replace biopsy or excision when feasible.

## 5 Discussion

### 5.1 Therapeutic efficacy in high-risk patients

Our three high-risk patients’ responses align with prior hedgehog inhibitor experience in laBCC. The phase II BOLT trial reported an ORR of 43% with sonidegib 200 mg daily (7). A Chinese study of 18 laBCC patients treated ≥6 months reported an ORR of 94% (78% complete, 17% partial, 6% stable) (4). All our patients improved with extended treatment, suggesting longer duration may yield additional benefit. However, clinical regression alone does not guarantee deep histological clearance, a distinction central to the imaging correlations below.

### 5.2 Ultrasound monitoring and imaging–pathology correlation

Post-sonidegib dermoscopy can mislead: pigmentation, scarring, and inflammation may persist despite tumor inactivation. Even pCR patients may retain arborizing vessels (53.8%) or blue-gray dots (61.5%) (4); similar persistence occurs in 66.7% of facial BCC (8). In our series, blue-gray structures persisted in all cases at 4–6 months. These may reflect pigmented remnants, melanophages, or stromal remodeling, but viable tumor cannot be excluded without histology. Dermoscopy mainly visualizes epidermis/superficial dermis, potentially missing deeper residual disease (4). HFUS complements it by depth-resolved assessment of thickness, infiltration, morphology, and vascularity (6–22 MHz).

### 5.3 Clinical applications of HFUS

Deep-tissue response: In Case 2, 3-month dermoscopy showed scar-like areas, but HFUS found a 10.3 × 3.9 × 8.2 mm hypoechoic nodule with internal flow, preventing premature treatment cessation.

Blood flow as activity marker: In Cases 1 and 2, Doppler reduction/disappearance correlated with tumor suppression more sensitively than size. At 9 and 8 months, residual hypoechoic foci lacked flow; histology confirmed pCR with only inflammation/fibrosis. Absence of flow plus no suspicious dermoscopy made inactivation probable. However, Case 3 shows that vascular regression still warrants continued treatment if dermoscopic suspicion persists.

Internal structure: Pretreatment anechoic areas in Case 3 resolved after 6 months, indicating stromal regression.

pCR prediction: In Cases 1 and 2, complete imaging response—ill-defined margins, no hyperechoic foci, no Doppler flow, scar-like dermoscopy—accurately predicted pCR. Concordant disappearance on both modalities may signal high pCR probability, though only two cases.

### 5.4 Very-high and ultra-high frequency ultrasound

Conventional HFUS (15–20 MHz) assesses thickness, depth, and vascularity. Very-high-frequency ultrasound (VHFUS; 30–100 MHz) and ultra-high-frequency ultrasound (UHFUS; >100 MHz) provide higher resolution and may differentiate postoperative fibrosis from residual tumor. Residual/recurrent tumors often appear irregular, ill-defined, heterogeneous, hypoechoic, and hypervascular on color Doppler, whereas fibrosis is regular, well-defined, homogeneous, and hypovascular. However, active fibrosis or inflammation can mimic residual tumor, and VHFUS/UHFUS penetration is limited to a few millimeters. Even UHFUS gives false positives in fibroblast-rich remodeling, and evidence comes mainly from melanoma scars, not BCC (9). Thus, these findings require caution, and histopathology remains the gold standard.

### 5.5 Absent doppler signals

Absent Doppler signals do not necessarily demonstrate absence of viable tumor. Technical factors, small vessel caliber, slow flow, and limited Doppler sensitivity can cause false-negative findings. Therefore, absent Doppler flow alone cannot exclude residual viable tumor or replace histopathology. In two surgically treated cases, disappearance of Doppler flow coincided with pCR, suggesting a promising imaging marker of treatment response. However, this hypothesis requires validation in larger cohorts before it can be used to defer biopsy. Until then, decisions should integrate dermoscopic, ultrasound, and clinical findings, as in Case 3.

### 5.6 Technical factors

Low-velocity flow detection depends on transducer frequency, gain, wall filter, pulse repetition frequency, lesion depth, and probe pressure. Excessive pressure can collapse small vessels. A gel stand-off pad increases transducer-skin distance, placing superficial lesions in the focal zone, reducing reverberation and compression, and improving detection of peri- and intralesional flow (10). Standardized settings, minimal pressure, and routine stand-off pads should be considered.

### 5.7 Strengths and limitations

Strengths include longitudinal multimodal assessment (clinical, dermoscopy, HFUS, histology in two cases) in high-risk laBCC. HFUS prevented premature cessation and predicted pCR in two confirmed cases, adding value over dermoscopy at facial sites.

Limitations: small sample (n=3), single-operator flow-detection bias, retrospective design, and absent histology in Case 3. Grayscale ultrasound cannot reliably distinguish fibrosis/inflammation from microscopic residual tumor; lesions ≤1–2 mm may be undetectable. Satisfactory imaging response cannot exclude microscopic disease, and flow disappearance should not be equated with sterilization. Our protocol lacked stand-off pads and higher-frequency transducers, reducing sensitivity to low-velocity flow.

## 6 Conclusion

Combining HFUS with dermoscopy offers a noninvasive approach for monitoring BCC response to sonidegib. HFUS complements dermoscopy by assessing deep architecture, vascularity, and regression; loss of intralesional Doppler flow suggests reduced tumor activity. In two surgical patients, complete imaging response (marked regression, no vascularity, resolved dermoscopic malignancy) correlated with pathological complete response. However, posttreatment hypoechoic areas may reflect inflammation, fibrosis, or residual tumor, limiting specificity. Thus, imaging-based complete response should be confirmed by biopsy or excision when feasible. Non-surgical patients need close clinical, dermoscopic, and ultrasound follow-up; surgical candidates still require excision and histopathology as the gold standard. Future larger, blinded, multi-operator studies with standardized Doppler protocols (stand-off pads), quantitative flow analysis, and very-high/ultra-high frequency ultrasound are needed to validate noninvasive criteria; long-term follow-up remains essential.

## Data Availability

The datasets presented in this study can be found in online repositories. The names of the repository/repositories and accession number(s) can be found in the article/supplementary material.
